# High capacity gas capture and selectivity properties of triazatruxene-based ultramicroporous hyper-crosslinked covalent polymer

**DOI:** 10.3906/kim-2102-70

**Published:** 2021-06-30

**Authors:** Ali Enis SADAK

**Affiliations:** 1 Chemistry Group Laboratories, TUBITAK National Metrology Institute (NMI), Kocaeli Turkey

**Keywords:** Friedel-Crafts, IAST (ideal adsorbed solution theory), microporous organic polymer, triazatruxene, hyper-crosslinked polymers, gas uptake

## Abstract

Tuning the selective sorption features of microporous organic networks is of great importance for subsequent applications in gas uptake and hiding, while it is more attractive in terms of being both time and cost effective to realize these optimizations without using functional groups in the core and linker. “Knitting” is one of the easiest and most used method to obtain a broad scope of hyper-crosslinked polymers on a large scale from aromatic structures that do not contain functional groups for polymerization. By the use of Knitting method, a hypercrosslinked covalent ultramicroporous organic polymer was obtained via stepwise process from using triazatruxene (TAT) as core -a planar indole trimer- through anhydrous FeCl_3_ catalyzed Friedel–Crafts alkylation using dimethoxybenzene as a linker. The resulting microporous polymer, namely TATHCCP was completely identified by analytical and spectral techniques after examined for gas properties (CO_2_, CH_4_, O_2_, CO, and H_2_) and selectivity (CO_2_/N_2_, CO_2_/O_2_, for CO_2_/CO and CO_2_/CH_4_) up to 1 bar and increased temperatures (273 K, 296 K and 320 K). Although it has a relatively low (Brunauer–Emmett–Teller) BET specific surface area around 557 m^2^/g, it was seen to have a high CO_2_ capture capacity approaching 10% wt. at 273 K. In accordance with (ideal adsorbed solution theory) IAST computations, it was revealed that interesting selectivity features hitting up to 60 for CO_2_/N_2_, 45 for CO_2_/O_2_, 35 for CO_2_/CO, 13 for CO_2_/CH_4 _at lower temperatures revealed that the material has much better selectivity values than many HCP (hyper-crosslinked polymer) derivatives in the literature even from its most similar analog dimethoxymethane derivative TATHCP, which has a surface area of 950 m^2^/g.

## 1. Introduction 

Except for a small portion of the energy currently used worldwide, such as 13%, the remainder is obtained by using fossil fuels. As a result of this high energy need, which causes climate change with high CO_2_ emissions, a wide variety of CO_2_ capture and separation techniques are being developed. In addition to the chemical CO_2_ capture process of the currently used amine solutions (diluted with 70% water) that are toxic, corrosive and degradation over time, the highly porous recyclable polymers that work according to the physisorption process are highly promising [1]. By increasing the surface area and addition of electron rich atoms on these materials, CO_2_ uptake and selectivity are increased, as well as the capture affinity for other pipe gases (a pipe gas includes SO_x_ (<800 ppm), NO_x_ (<500 ppm), O_2_ (3%–4%), H_2_O (5%–7%), CO_2_ (15%–16%) and N_2 _(75%–76%)) can be practically changed [2]. Increased hydrogen bonding ability and dipole quadrupole interactions by using polar groups such as amine, hydroxyl and halogen of the porous material surface can capture CO_2_ gas more selectively on N_2_ and CH_4_ [3]. Accordingly, synthesis of new porous materials using nitrogen-rich monomers and combination of polar groups into their construction through post modification techniques are of great interest [4,5]. Microporous organic polymers consisting of light nonmetallic elements with large surface area, narrow pore size, and high degree of thermal and chemical stability are extremely low-cost cutting-edge materials used for gas sorption and storage processes. Examples of many microporous polymers with high gas capture and separation capacities include triazine derivative crystalline organic networks (CTFs) [6,7] , covalent organic networks (COFs) [8], microporous conjugated polymers (CMPs) [9,10], intrinsic microporosity polymers (PIMs) [11,12], porous aromatic frameworks (PAFs) [13] and hypercrosslinked polymers (HCPs) [14–17] can be given. Hyper-covalent conjugated polymers (HCCPs), another subclass of MOPs, differ from HCPs due to their conjugation. Although the synthesis methods are similar to HCPs, it has been found that HCCPs are more effective especially in gas selectivity thanks to their continuous conjugation due to the use of aromatic linkers in their synthesis [14]. Triazatruxene, which contains three nitrogen atoms that highly effective in gas capture processes, is an important indole derivative due to its planar and aromatic structure, as well as active benzene rings in Friedel–Crafts alkylation reaction. Triazatruxene derivatives have a great interest as a result of their high electron supply capability provided by the p-conjugate structure with high electron mobility and thermal stability provided in complex structures [18–22]. It was observed by our group that the HCP derivative of triazatruxene (TATHCP) synthesized by using the methylal linker has a high degree of gas adsorption and selectivity [15]. Therefore, in this study in order to see how change the gas uptake and selectivity properties of TATHCP by the change of linker, the synthesis, gas uptake and selectivity properties of the hypercovalent conjugated structure TATHCCP was obtained by using dimethoxybenzene as linker and triazatruxene as core. Despite the lower surface area of TATHCCP compared to its analog TATHCP, there is notable increase of CO_2_ selectivity over N_2_, CH_4_ and CO of TATHCCP, which shows the obvious difference on the use of aromatic linker in the selectivity.

## 2. Materials and methods

### 2.1. Materials

POCl_3_ 99% purity was purchased from Sigma-Aldrich (Sigma-Aldrich Corp., St. Louis, MO, USA). 2-oxoindole 97% purity was procured from Sigma-Aldrich. Dimethoxymethane reagent plus 99% purity was purchased from Sigma-Aldrich. KOH ≥ %85 purity was purchased from Merck. All materials were used as received, unless otherwise stated.

### 2.2. 10,15-Dihydro-5H-diindolo[3,2-a:3’,2’-c]carbazole (Triazatruxene, 2)

In a 50 mL round-bottomed flask there was added 2-oxoindole (1; 2 g, 15 mmol) into POCl_3_ (10 mL, 105 mmol) stirred until dissolved at RT then stirred at 100 °C for 8 h. After 8 h, reaction mixture was cooled to room temperature and poured into a 500 mL beaker containing ice chips (250 mL) and saturated KOH was added until the pH value reached 7. The resulting dark green colored settlings were collected by vacuum filtration using No. 2 sintered glass filtrate and the raw product (1.2 g) was purified by silica gel (150 g) column chromatography by using 4:1 ethyl acetate/hexane as the eluent. After crystallization from 4:1 acetone/hexane, 10,15-dihydro-5 *H* -diindolo[3,2- *a* :3’,2’- *c* ]carbazole (2) was obtained (Yield: 40%, 0.8 g) [23]. Melting point: 393-394 °C. ^1^H-NMR (600 MHz, DMSO): d 11.86 (bs, 3H), 8.67 (d, *J* = 7.6 Hz, 3H), 7.73 (d, *J* = 7.6 Hz, 3H), 7.40 – 7.32 (m, 6H). APT ^13^C-NMR (150 MHz, DMSO): d 139.0, 134.2, 123.0, 122.7, 120.3, 119.5, 111.4, 101.0. IR (KBr, cm^–1^): 3473, 3439, 3053, 3025, 2919, 2852, 1737, 1635, 1273, 729.

### 2.3. 5,10,15-triethyl-10,15-dihydro-5H-diindolo[3,2-a:3’,2’-c]carbazole (3)

In a 100 mL anhydrous THF, triazatruxene (2; 1.85 g, 5.36 mmol) and KOH (4.51 g, 80.34 mmol, 15 eq.) was added at room temperature and mixture was heated at 70 °C for 3.5 h. After cooling room temperature, ethyl bromide (2.33 g, 21.42 mmol, 4eq.) was added to the mixture. The mixture was stirred magnetically overnight at room temperature. After checking with TLC and understanding that the reaction was complete, solvent was removed under reduced pressure. The raw product was dissolved in 150 mL of EtOAc and washed with diluted NaHCO_3_ (1× 100 mL) then water (3 × 100 mL) and dried over MgSO_4_.The solvent was removed under reduced pressure. The raw product was purified on silica gel (20 g) column chromatography by using 1:4 CH_2_Cl_2_/hexane. After crystallization over acetone, 5,10,15-triethyl-10,15-dihydro-5H-diindolo[3,2-a:3’,2’-c]carbazole (3) was obtained. (Yield: 89%, 2.1 g) ^1^H-NMR (600 MHz, CDCl_3_): δ ppm 8.37 (d, J = 8.0 Hz, 3H), 7.68 (d, J = 8.0 Hz, 3H), 7.48 (t, J = 7.4 Hz, 3H), 7.37 (t, J = 7.4 Hz, 3H), 5.05 (t, J = 7.2 Hz, 6H), 1.64 (t, J = 7.2 Hz, 9H). ^13^C-NMR (150 MHz, CDCl_3_): δ ppm 143.4, 141.4, 126.2, 125.4, 124.1, 122.5, 113.0 105.9, 44.4, 18.2. IR (powder, cm^-1^): 3045, 2970, 1555, 1480, 1320, 1233, 1098, 724. HRMS: m/z: Calcd. for (C_30_H_27_N_3_) [M+H^+^]: 430.22385; found, 430.22868.

### 2.4. Synthesis of TATHCCP

In 20 mL nitrobenzene, 5,10,15-triethyl-10,15-dihydro-5H-diindolo[3,2-a:3’,2’-c]carbazole (3) (0.250 g, 0.582 mmol) and *p* -dimethoxybenzene (1.210 g, 8.73 mmol, 15 equiv.) in 20 mL 1,2-dichloroethane, anhydrous FeCl_3_ (4.150 g, 25.61 mmol, 44 equiv.) was added at room temperature. The mixture was stirred at 80°C for 5 h then at 120°C for 24 h under an inert (N_2_) atmosphere. After 24 h, reaction mixture was cooling to room temperature then the dark brown precipitate was collected by using No. 1 sintered glass filtrate and repeatedly washed with methanol, concentrated HCl, distilled water, and methanol to eliminate unreacted monomers and FeCl_3 _till the filtrate was almost colorless. Then, the TATHCCP was purified by Soxhlet extraction from THF (50 mL) for 24 h then dried under vacuum at 120 °C for 24 h to give dark brown-colored solid powder (Yield: 551 mg, 97 %). IR (powder, cm^-1^): 2927, 1572, 1457, 1427, 1324, 1208, 1135, 852, 811. 

## 3. Results and discussion

### 3.1. Synthesis and characterization

**Figure 1:**
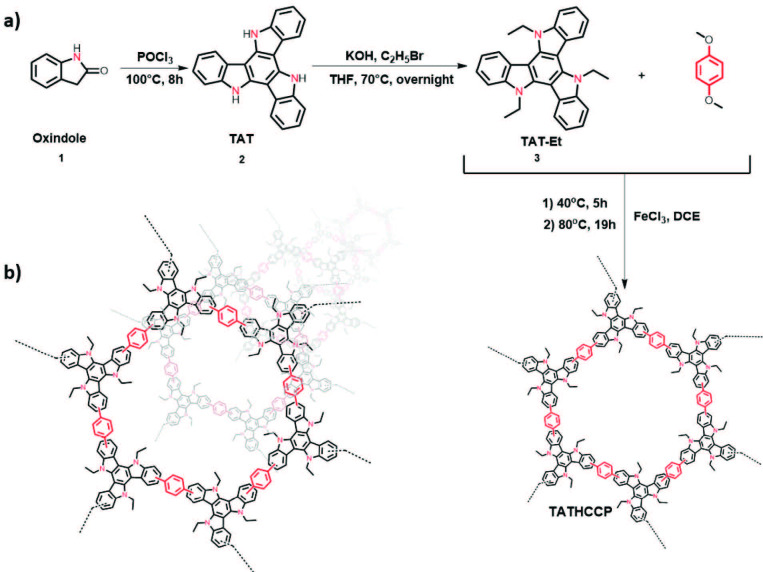
a) Synthesis of TAT-HCCP. b) Proposed structure of TAT-HCCP (the actual structure can be much more complex due to the dense crosslinking).

One of the easiest methods to obtain a broad diversity of hyper-crosslinked polymers on a large scale by using aromatic structures that do not including active groups for polymerization is known as the “knitting” method [24,25]. Here, a new hyper-crosslinked covalent polymers network called TATHCCP is introduced, which obtained with Friedel-Crafts alkylation using triazatruxene (TAT) as the core and dimethoxymethane as the external linker (Figure 1a). Like the other similar HCPs synthesized using the same knitting method, the yield of TATHCCP was also quantitative [24,26]. The triazatruxene ring was obtained in a similar way to the previous work [23], and N-alkylation of the nitrogen atoms of the trimer structure was performed with ethyl bromide to increase the solubility (Figure 1a). By increasing the equivalent amounts of catalyst from 3 equivalents to 40 equivalents, the surface area of the obtained TATHCCP increased from 40 m^2^/g to 557 m^2^/g. The stability of the obtained TATHCCP polymer in water and most organic solvents even in diluted NaOH and HCl simplified the purification and activation steps.

### 3.2. Textural and spectral properties

**Figure 2:**
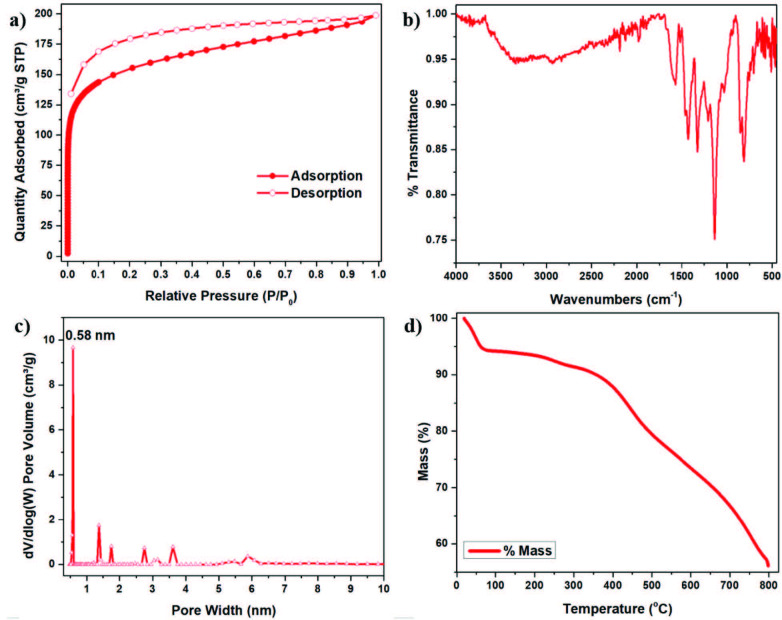
Structural characterization of TATHCCP (a) N2 adsorption-desorption isotherms at 77 K, (b) FT-IR spectrum, (c) pore size distribution, (d) thermogravimetric analysis.

**Figure 3:**
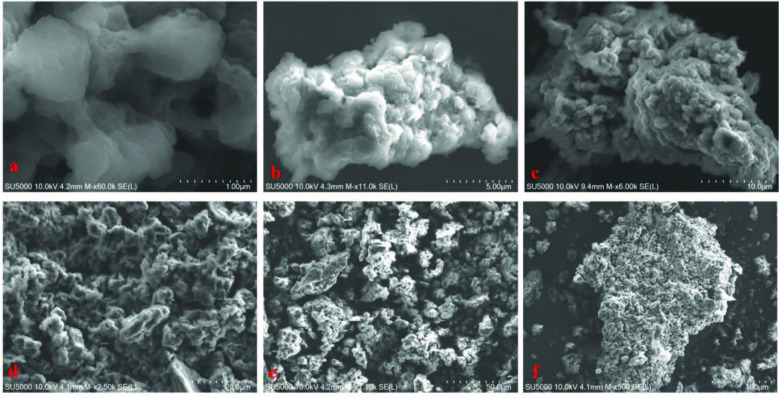
SEM images of TATHCCP (a–f). See SI for more images.

FT-IR, SEM, TEM, XRD, and solid-state magic angle spinning (CP/MAS) ^13^C NMR spectrometry were used to examine of the spectral properties of obtained material, as shown in Figure 2 and Figure S1-S10, SI. FT-IR peaks show the C−N−C moieties appear as characteristic bands at around 1450 cm^−1^ while aromatic C=C bands (stretching) vibrations around 850–1580 cm^–1^ which belong to carbazole pyrrole fused benzene rings and crosslinked benzene moieties. C−H vibrations (stretching) around 2927 cm^–1^ indicate that the material is appropriate with the supposed cross-linked polymer (Figure 2). ^13^C CP-MAS NMR spectroscopy was utilized to further support the chemical structure of the TATHCCP (Figure S1, SI). Peaks resonance at 143 and 127 ppm belong to fused carbazole rings of TAT, while the peak near 57 ppm belongs to carbon in unreacted methoxy group of dimethoxybenzene crosslinker showing the uncompleted reaction of TATHCCP. In addition, unsubstituted aromatic carbons of dimethoxybenzene have resonance at 157 ppm while the peak of the carbon atoms at the 3-position of the indole trimer a resonance at about 106 ppm (Figure S1, SI). Asterisks denote at 217, 188, 117, and 95 ppm are denoted spinning sidebands. SEM and EDX analysis was examined for particle composition and purity of the TATHCCP polymer. From SEM images of polymer, the findings suggest formation of submicrometer size of aggregated particles, while EDX analysis showed that there was not FeCl_3_ catalyst in the polymer obtained, except for C and N elements and O element from unreacted dimetoxymethane moiety. (Figure 3, SI). The powder XRD spectrum has not indicate any typical peaks showing that the polymer has greatly amorphous character (Figure S2, SI). 

It is seen from the thermogravimetric analysis (TGA) spectrum that the polymer has a thermal stability up to 350 °C, and it is also seen that it retains 50% of its mass up to 800 °C (Figure 2d). Possible reason of this significant retain from the mass of TATHCCP is N-doped carbon because of thermal disintegration. Because of residuary solvent and moisture evaporation there is an initial weigh loss near at 90 °C. Considering the results of the TGA analysis, it is seen that TATHCCP is a possible candidate as it meets the requirements in high temperature processes such as CO_2_ capture and post combustion.

### 3.3. Porosity

Surface area and porosity of TATHCCP network were characterized at 77 K by using N_2_ as probe gas. Activation of the polymer was achieved by degassing for 12 h at 200 °C to start the analysis. As can be seen from the N_2_ adsorption desorption isotherm in Figure 2a, the sharp uptake at the initial low relative pressure (0−0.1 = P/P_o_) region and above 0.1 at higher relative pressure shows that TATHCCP has a highly microporous character as well as a permanent microporosity. Considering the IUPAC [27] classification, it is seen that TATHCCP exhibits mixture of type I and V isotherm identity at increased pressures (Figure 2a). TATHCCP shows a mild capillary condensation between P/P_o_ 0.9−0.95 that is commonly presented in microporous networks owing to distension force of the structure throughout the touch of interstitial gaps by the adsorbate molecules in the network [28]. The lack of edged adsorption peak at 0.8−1.0 P/P_o_ high relative pressure area that points out the macroporous character reveals that TATHCCP doesn’t have this kind of pore structures (Figure 2a). Calculated surface area analysis from the Langmuir (S Lang) and BET (S_BET_) (Brunauer – Emmett – Teller) theories and textural properties are given in Table 1. According to Langmuir model, surface area of the TATHCCP was found 702 m^2^g^–1^, while it was found 557 m^2^g^–1 ^for Brunauer – Emmett – Teller model as shown in Figures S3, SI and Figures S4, SI. The findings taken from pore size distribution graph (PSD) by using the nonlocal density functional theory (NLDFT) showed that the network has immensely (> 60%) ultramicropore character at 0.6 nm that promising for gas uptake and selectivity processes (Figure 2c).

**Table 1:** The textural properties of the TATHCCP.

Polymer	SBETa[m2 g–1]	SLangb[m2 g–1]	Smicroc[m2 g–1]	Vtd[cm3 g–1]	V0.1e(cm3 g–1)	%V0.1/Vt	%Smicro/SBET	4V/AfBET[nm]
TATHCCP	557	702	345	0.31	0.26	84	62	2.20

a. BET surface area calculated from N2 adsorption isotherm in the relative pressure (P/P0) range from 0.05 to 0.20. b. Langmuir surface area calculated from N2 adsorption isotherm in the pressure range from 30 to 220 mbar. c. Micropore surface area calculated from the N2 adsorption isotherm using t-plot method from the Harkins–Jura equation. d. Total pore volume at P/P0 = 0.99. e. Micropore volume at P/P0 = 0.1. f. Average pore diameter.

Total pore and micropore volume of TATHCCP was found 0.31 and 0.26, respectively at relative pressures P/P0 = 0.99 and 0.1. By using the ratio of micropore volume to total pore volume, degree of microporosity of TATHCCP was calculated as 84%, which is quite high compare with it’s before synthesized hyper covalent analog TATHCP (%70).

### 3.4. Gas uptake and selectivity

**Figure 4:**
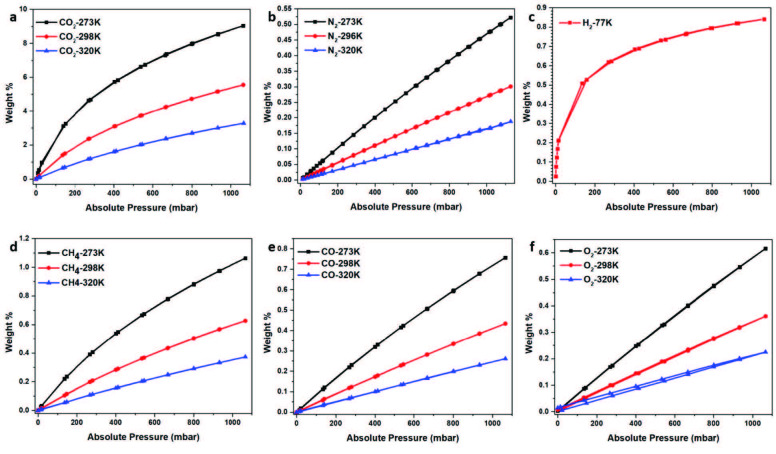
Gas uptake (weight %) properties of TATHCCP. (a) CO2, (b) N2, (c) H2, (d) CH4, (e) CO, (f)O2 CO adsorption-desorption isotherms at 273K, 298K and 323 K.

The amazing degree of microporosity (83%) with high amount of electron rich nitrogen bones of TATHCCP was encouraging to examine comprehensively for the possibility of promising gas uptake properties. Five different gas including CO_2_, N_2_, CH_4_, CO, and O_2_ was used to determine the adsorption properties of TATHCCP from 0 to 1.1 bar pressures at 273 K, 298 K, and 320 K (Figure 4, Table S1). CO_2_ uptake capacity of TATHCCP was found 9.0% by weight at 273K /1.1 bar, 5.5% by weight at 298 K /1.1 bar, and 3.2% by weight at 298K /1.1 bar, respectively (Figure 4a) . CO_2_ physisorption process seems reversible as the desorption isotherms are very close to adsorption isotherms. Although TATHCCP has a moderate surface area with respect to its hyper covalent analog TATHCP, obtained CO_2_ uptake values are very close to TATHCP at all temperature, and higher or closer than lots of networks with nearly equal surface area in literature (Table S3) [1, 29–34]. Main effect of this high CO_2_ adsorption capacity of TATHCCP is due to the lone pair electrons of high amount nitrogen atoms on network, which are used to catch CO_2_ molecules with hydrogen bonding. In contrast to CO_2_ adsorption values, N_2_ adsorption values of TATHCCP were found very low at all three temperatures, which can be summarized as the TATHCCP has highly N_2_ phobic character. N_2_ uptake capacity of TATHCCP was found 0.52 wt%, 0.30 wt% and 0.18 wt% at 273 K, 298 K and 320 K at 1.1 bar, respectively (Figure 4b). 

The Clausius–Clapeyron equation was used to obtain the isosteric heat of adsorption ( *Q*_st_) values of TATHCCP to determine whether CO_2_ is physically adsorbed. Calculated *Q*_st_ values of TATHCCP was found 34.7 kJ mol **^–^** ^1^ at zero loading and the fact that it remained as 29.5 kJ mol **^–^** ^1^ even when the adsorbed amount of CO_2_ increased reveals that the network has well retention ability despite the low surface area (Figure S8, SI). Additionally, TATHCCP physically adsorbs CO_2_ molecules due to the *Q*_st_ values not more than 50 kJ mol **^–^** ^1^, and thanks to the weak interactions with CO_2_, the network has a good recycle ability, which needs no more energy. Compared to low pressure and compressed form, physically adsorbed CH_4_ storage utilities are safer for long transportation processes and in terms of cost. Moreover, in clean energy applications, the physically adsorption of hydrogen is of great interest. Even if much higher pressures are needed to determine the exact gas uptake values for H_2_ and CH_4_ adsorption, low pressure uptakes up to 1 bar help to see latter gas separation utilities. Therefore, the adsorption properties of TATHCCP were tested by using H_2_ at 77 K and CH_4_ at 283, 298, and 320 K as probe gases. H_2_ adsorption value of TATHCCP was found 0.84 wt % at 77 K, while CH_4_ adsorption values was found 1.06 wt%, 0.62 wt% and 0.37 wt% at 283, 298, and 320 K at 1.1 bar separately (Figures 4c, 4d). It is understood that higher H_2_ capacity can be obtained at higher pressures as saturation cannot be reached as seen from the H_2_ isotherm of TATHHCCP performed at 77K. Also, this H_2_ uptake values are remarkable when compared with previous works [35–38] (Table S4). The obtained CH_4_ uptake values of TATHCCP are comparable with the previous works [28, 36, 39–45] (Table S5). Also, the calculated *Q*_st_ values of CH_4_ by using isotherm data at 273 and 298 K (Figure S9) indicate that TATHCCP has 23.31 kJ mol^−1^ at zero coverage. Difference between the *Q*_st_ values of CH_4_ and CO_2_ is due to the nonpolar character of CH_4,_ which has a less interaction with polymer compared with CO_2_ molecules.

TATHCCP adsorption capacity was also examined by using CO and O_2_ as probe gases separately at 273 K, 298 K, and 320 K at 1.1 bar before calculation of selectivity properties. CO adsorption values of TATHCCP was found 0.75 wt%, 0.43 wt%, 0.26 wt% at 273, 298, and 320 K while O_2_ uptake values was found 0.61 wt%, 0.36 wt%, and 0.22 wt% at 273, 298, and 320 K at 1.1 bar, respectively (Figures 4e, 4f).

**Figure 5:**
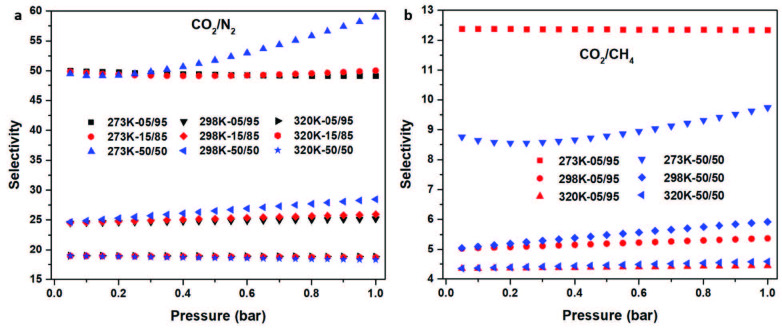
Selectivity calculations of TATHCCP using the IAST for (a) CO2/N2-05/95, 15/85, and 50/50 at 273 K, 296 K, and 320 K, (b) CO2/CH4-05/95 and 50/50 at 273 K, 296 K, and 320 K.

**Figure 6:**
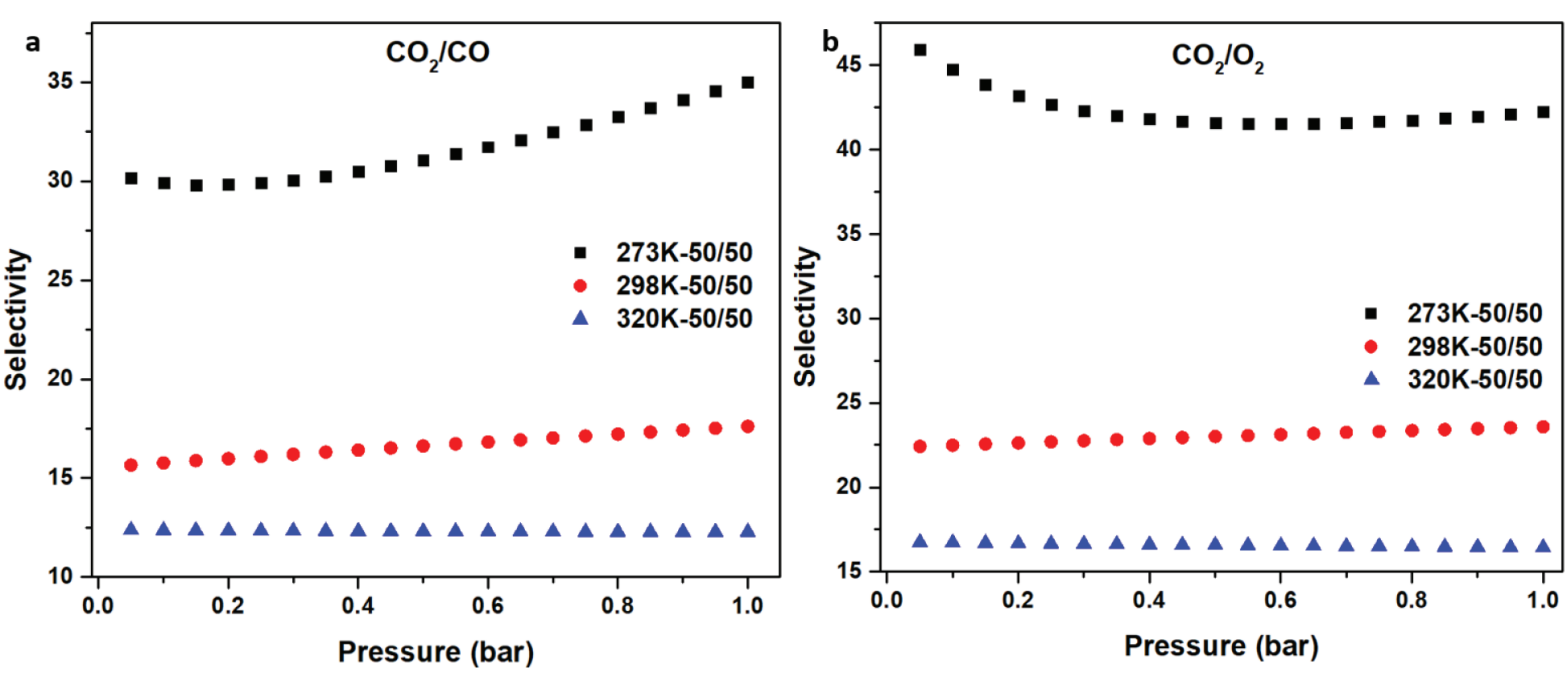
Selectivity calculations of TATHCCP using the IAST for (a). CO2/CO-50/50 at 273 K, 296 K and 320 K. (b). CO2/O2-05/95 and 50/50 at 273 K, 296 K, and 320 K.

Besides, high CO_2_ sorption values, recycle ability, and CO_2_ selectivity over other pipe gases have the most important subjects of a new porous material candidate for using post combustion processes. Myers and Prausnitz’s ideal adsorbed solution theory (IAST) is one of the most used technique for prediction of adsorption feature from using one-ingredient gas isotherms to calculate multi-components [46]. IAST was used to compute the CO_2_ selectivity properties of TATHCCP over four different gases (CH_4_, N_2_, CO, and O_2_) at the three different temperatures (273, 298, and 320 K) used for adsorption analysis up to 1 bar (Figures 5, 6 and Table S2). To see the minor differences, CO_2_/N_2_ selectivity of TATHCCP was calculated at three different pipeline ratios (05/95, 15/85, and 50/50) (Figures 5a, 5b). Additionally, CO_2_/CH_4_ selectivity of TATHCCP was calculated at two different ratios (05/95 and 50/50) while CO_2_/CO and CO_2_/O_2_ selectivities were calculated at 50/50 ratio (Figure 6a, b). CO_2_/N_2_ selectivity values of TATHCCP were found 59.1, 28.5, and 18.3 at 50/50 ratio, while they were found 50.0, 25.9, 18.3 at 15/85 ratio and 49.2, 25.9, 18.2 at 05/95 ratio at 273 K, 298 K and 320 K at 1.1 bar pressure, respectively (Figure 5a). Obtained selectivity values of TATHCCP at three different ratios and temperatures are close to each other and higher than previous networks in literature and especially its hypercovalent analog TATHCP [47–51] (Table S2, S6, SI). 

Selectively separation of CO_2_ from CH_4_ is very important issue in the purification process of natural gas due to preventing of pipeline corrosion and rise of obtained energy yield [28]. CO_2_/CH_4_ selectivities of TATHCCP at 50/50 ratio were found 12.3, 5.4, and 4.4, while they were found as 9.7, 5.9, and 4.5 at 05/95 ratio at 273 K, 298 K, 320 K at 1.1 bar pressure, respectively (Figure 5b). Obtained CO_2_/CH_4_ selectivities at 05/96 ratio of TATHCCP are nearly the same of its analog TATHCP at all three degrees and higher than most of the previous works at 273 K (Table S7) [47,52,53]. 

CO_2_/CO and CO/O_2_ selectivities of TATHCCP were also calculated at the same temperatures and pressure as to compare to TATHCP and previous works. Calculated CO_2_/CO selectivities of TATHCCP were found as 35.0, 17.6, and 12.3 at 50/50 ratio at temperatures 273, 296, and 320 K at 1.1 bar, respectively while CO_2_/O_2_ selectivies were found 45.9, 23.5, and 16.4 at the same temperatures and pressure (Figures 6a, 6b). Both of CO_2_ selectivities over CO and O_2_ of TATHCCP at 273 K are higher than its analog TATHCP while a little low at other two temperature (Table S2). When TATHCCP (557 m^2^/g) and TATHCP (997 m^2^/g) obtained from the same triazatruxene core are compared considering the selectivity results, its obviously seen that specific surface area is not the main effect of selectivity. Continuous resonance of TATHCCP is one of the possible reasons of high selectivity values than TATHCP, and the other one is probably due to higher degree of microporosity of TATHCCP (%84) compared to TATHCP (%70), which helps to more selective CO_2_ catching. These differences in selectivity and sorption properties observed between these two hypercovalent polymers based on triazatruxene cannot be generalized for all hypercovalent polymers that consist of the same core but contain only differentiated linkers. Each polymer obtained with high porosity has its own selectivity and sorption properties arising from the core from which it is formed. It’s known from previous studies of HCP derivatives that even if the change of linkers affects the selectivity properties, the core of the polymer and the different atoms attached to the core also affect the selectivity values of the obtained polymers [14,54]. Therefore, regardless of whether the linkers used in different studies are the same, if the core used is different from the one used in the other study, the selectivity and sorption properties should be examined from the beginning. In this way, changes can be observed much more clearly by both the linker and the core. This study has been one of the rare studies in which these differences are shown specifically for linkers.

## 4. Conclusion

In sum, starting from electron-rich triazatruxene and using aromatic dimethoxybenzene ring as a linker, a new microporous hypercrosslinked conjugated polymer, TATHCCP, was synthesized by using FeCl_3_ catalyzed Friedel–Crafts reaction. The high degree of micropore character of 84%, together with the moderate surface area, prompted the study of TATHCCP’s gas uptake and selectivity properties. The gas uptake properties are comparable to most of the similar structures in the literature, as well as higher selectivity properties than many of the previously synthesized HCPs and especially its previous analog TATHCP, revealed that TATHCCP has a high potential for CO_2_ separation processes in flue gas systems. The obtained *Q*_st_ values in the physical adsorption region for CO_2_ and CH_4_, its resistance to highly acidic-basic environments and different solvents combined with its cheap structure make TATHCCP very useful for industrial applications. Furthermore, this study showed how the degree of microporosity also plays an important role, as well as the effect of high specific surface area on selectivity properties. 

Supplementary Materials Click here for additional data file. Materials and methods, synthesis, Langmuir and BET area plots, scanning electron microscopy (SEM) images, energy-dispersive X-ray spectroscopy (EDS) images and spectra, solid-state ^13^C CP-MAS NMR spectra, adsorption selectivities of CO_2_ over N_2_, CH_4_, O_2_, CO, tables of selectivity, gas adsorption capacity and CO_2_, CH_4_, H_2 _uptakecomparison of different microporous materials.
