# Supplementary material for: High capacity gas capture and selectivity properties of triazatruxene-based ultramicroporous hyper-crosslinked covalent polymer
Source: Turk J Chem. 2021 Jun 30;45(3):868–78. doi: 10.3906/kim-2102-70 (PMC8326483; doi:10.3906/kim-2102-70)
Supplement: Supplementary file 1 — Supplementary Materials [file turkjchem-45-868-sup001.pdf]

Supporting Information

Content Page

1. General methods and characterization……………….……………………………………………....2
2. Synthetic procedures……………………...………………………………………………………....2
2.1. 10,15-Dihydro-5H-diindolo[3,2-a:3',2'-c]carbazole (Triazatruxene, 2)……………..…...2
2.2. 5,10,15-triethyl-10,15-dihydro-5H-diindolo[3,2-a:3',2'-c]carbazole (3) ………….…..3
2.3. Synthesis of TATHCCP…………………………...……………………….…………..…3
3.General Spectra of TATHCCP…………………………...……………………….……...…………..4
Figure S1. (CP/MAS) ^{13}C NMR spectra of TATHCCP………………………….....................4
Figure S2. PXRD pattern profile of TATHCCP…..…………………………………………….…4
Figure S3. Langmuir surface area plot for TATHCCP calculated from the isotherm...............5
Figure S4. BET surface area plot for TATHCCP calculated from the isotherm ……….…..…5
Table S1. Comparable gas adsorption capacity of the TATHCCP with TATHCP....................6
Table S2. Comparable results of gas selectivity for TATHCCP with TATHCP.............……..6
Table S3. Comparison of different microporous materials with respect to their textural and CO_2 uptake (wt % - mmol) values……………………………………………………………….…....6
Table S4. Comparison of different microporous materials with respect to their H_2 uptake (wt %) values.................................………..7
Table S5. Comparison of different microporous materials with respect to their CH_4 uptake (wt %) values.................................………..7
Table S6. Comparison of different microporous materials with respect to their CO_2/N_2 (15/85) selectivity values at 273 K…...7
Table S7. Comparison of different microporous materials with respect to their CO_2/CH_4 (50/50) selectivity values at 273 K…...8
Figure S5. Initial slope fitting for gases of TATHCCP at 273 K…………………...……………8
Figure S6. Initial slope fitting for gases of TATHCCP at 298K…………………………….…….9
Figure S7. Initial slope fitting for gases of TATHCCP at 320 K…...9
Figure S8. The isosteric heat of adsorption (Q_{st}) of TATHCCP for CO_2……………………...10
Figure S9. The isosteric heat of adsorption (Q_{st}) of TATHCCP for CH_4……………………...10
Figure S10. EDS spectra of TATHCCP..........……..11
4. Scanning Electron Microscopy (SEM) Images of TATHCCP..12
7. References................................ 14

1. General methods and characterization

All reagents were purchased from commercial supplier (Sigma-Aldrich Corp.) and used without further purification. Solid-state NMR measurements were carried out with Bruker Ascend 400 MHz spectrometer (Billerica, Massachusetts, ABD). The ^{13}C CP/MAS NMR spectra were obtained with a 4-mm double-resonance MAS probe and with a sample spinning rate of 8.0 kHz; a contact time of 2 ms and pulse delay of 3 s were acquired. The gas adsorption and desorption experiments were performed using Micromeritics 3Flex system. The samples were degassed at 200 °C for 12 h before the measurements. Surface areas were determined from the adsorption data using Brunauer–Emmett–Teller (BET) and Langmuir methods. The pore-size-distribution curves were obtained from the adsorption curve using nonlocal density functional theory (NLDFT) method. Scanning & Transmission Electron Microscopy (SEM & STEM) images were obtained Hitachi SU-5000 (Chiyoda, Tokyo, Japan) microscope worked at changing voltage of 5.0 – 30.0 kV. The thermal behaviour of polymer materials was investigated thermogravimetric analysis (TGA) instrument (Eixstar) over the temperature range of 20 to 800 °C under N_2 atmosphere. FTIR spectra were recorded with Bruker Alpha-P instrument. Powder X-ray diffraction (PXRD) results were obtained with Shimadzu XRD 6000 (Shimadzu Scientific Instruments Incorporated, Kyoto, Japan) operated at 40 kV and 40 mA with Cu Kα radiation (step size: 0.020, step time: 0.60 s).

2. Synthetic procedures

2.1. 10,15-Dihydro-5H-diindolo[3,2-a:3',2'-c]carbazole (Triazatruxene, 2)

In a 50 mL round-bottomed flask there was added 2-oxoindole (1; 2 g, 15 mmol) into $POCl_3$ (10 mL, 105 mmol) stirred until dissolved at RT then stirred at 100 °C for 8 h. After 8 h, reaction mixture was cooled to room temperature and poured into a 500 mL beaker containing ice chips (250 mL) and saturated KOH was added until pH value is 7. The resulting dark green colored settlings was collected by vacuum filtration using No. 2 sintered glass filtrate and the raw product (1.2 g) was purified by silica gel (150 g) column chromatography by using 4:1 Ethyl acetate/hexane as the eluent. After crystallization from 4:1 acetone/hexane, 10,15-dihydro-5*H*-diindolo[3,2-*a*:3',2'-*c*]carbazole (2) was obtained (Yield: 40%, 0.8 g)[23]. Melting point: 393–394 °C. ^{1}H-NMR (600 MHz, DMSO): δ 11.86 (bs, 3H), 8.67 (d, J = 7.6 Hz, 3H), 7.73 (d, J = 7.6 Hz, 3H), 7.40–7.32 (m, 6H). APT ^{13}C-NMR (150 MHz, DMSO): δ 139.0, 134.2, 123.0, 122.7, 120.3, 119.5, 111.4, 101.0. IR (KBr, cm^{-1}): 3473, 3439, 3053, 3025, 2919, 2852, 1737, 1635, 1273, 729.

2.2. 5,10,15-triethyl-10,15-dihydro-5H-diindolo[3,2-a:3',2'-c]carbazole (3)

In a 100 mL anhydrous THF, triazatruxene (2; 1.85 g, 5.36 mmol) and KOH (4.51 g, 80.34 mmol, 15 eq.) was added at room temperature and mixture was heated at 70 °C for 3.5 h. After cooling the room temperature, ethyl bromide (2.33 g, 21.42 mmol, 4eq.) was added to the mixture. The mixture was stirred magnetically overnight at room temperature. After checking with TLC and understanding that the reaction was complete, solvent was removed under reduced pressure. The raw product was dissolved in 150 mL of EtOAc and washed with diluted $NaHCO_3$ (1 × 100 mL) then water (3 × 100 mL) and dried over $MgSO_4$. The solvent was removed under reduced pressure. The raw product was purified on silica gel (20 g) column chromatography by using 1:4 CH_2Cl_2/hexane. After crystallization over acetone, 5,10,15-triethyl-10,15-dihydro-5H-diindolo[3,2-a:3',2'-c]carbazole (3) was obtained. (Yield: 89%, 2.1 g) ^{1}H-NMR (600 MHz, $CDCl_3$): δ ppm 8.37 (d, J = 8.0 Hz, 3H), 7.68 (d, J = 8.0 Hz, 3H), 7.48 (t, J = 7.4 Hz, 3H), 7.37 (t, J = 7.4 Hz, 3H), 5.05 (t, J = 7.2 Hz, 6H), 1.64 (t, J = 7.2 Hz, 9H). ^{13}C-NMR (150 MHz, $CDCl_3$): δ ppm 143.4, 141.4, 126.2, 125.4, 124.1, 122.5, 113.0 105.9, 44.4, 18.2. IR (powder, cm^{-1}): 3045, 2970, 1555, 1480, 1320, 1233, 1098, 724. HRMS: m/z: Calcd. for ($C_{30}H_{27}N_3$) [$M+H^+$]: 430.22385; found, 430.22868.

2.3. Synthesis of TATHCCP

In 20 mL nitrobenzene, 5,10,15-triethyl-10,15-dihydro-5H-diindolo[3,2-a:3',2'-c]carbazole (3) (0.250 g, 0.582 mmol) and *p*-dimethoxybenzene (1.210 g, 8.73 mmol, 15 equiv.) in 20 mL 1,2-dichloroethane, anhydrous $FeCl_3$ (4.150 g, 25.61 mmol, 44 equiv.) was added at room temperature. The mixture was stirred at 80 °C for 5 h then 120°C for 24 h under an inert (N_2) atmosphere. After 24 h, reaction mixture was cooling to room temperature then the dark brown precipitate was collected by using No. 1 sintered glass filtrate and repeatedly washed with methanol, concentrated HCl, and distilled water to eliminate unreacted monomers and $FeCl_3$ till the filtrate was almost colorless. After that the TATHCCP was purified by Soxhlet extraction from THF (50 mL) for 24 h then dried under vacuum at 120 °C for 24 h to give dark brown-colored solid powder (Yield: 551 mg, 97 %). IR (powder, cm^{-1}): 2927, 1572, 1457, 1427, 1324, 1208, 1135, 852, 811.

3. General spectra of TATHCCP

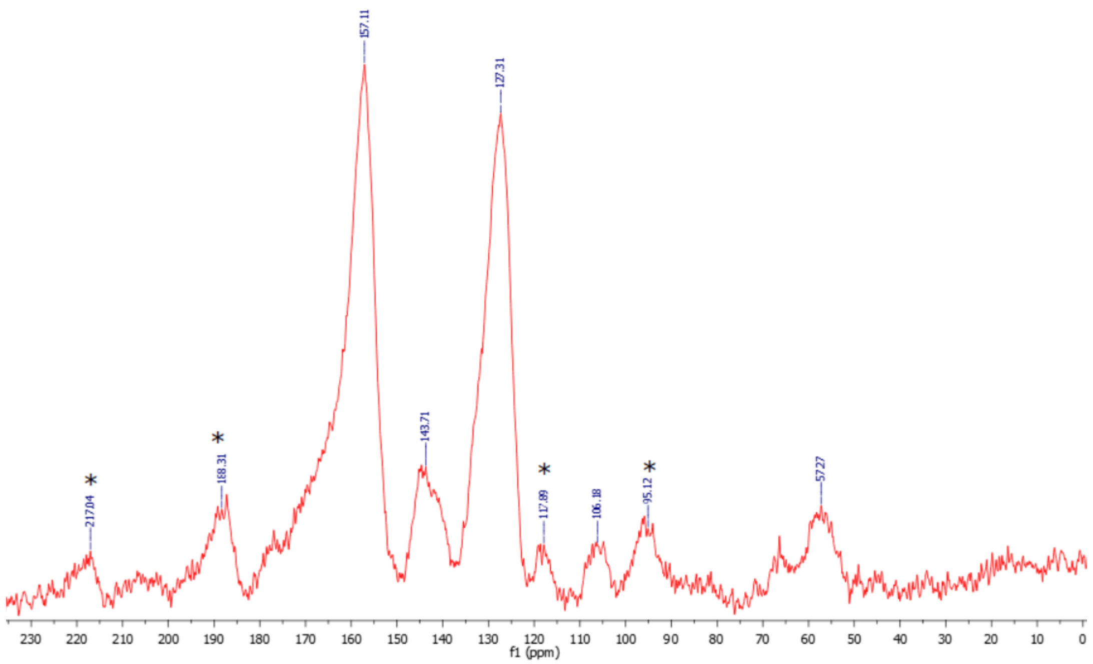

Figure S1. (CP/MAS) ^{13}C NMR spectra of TATHCCP**.**

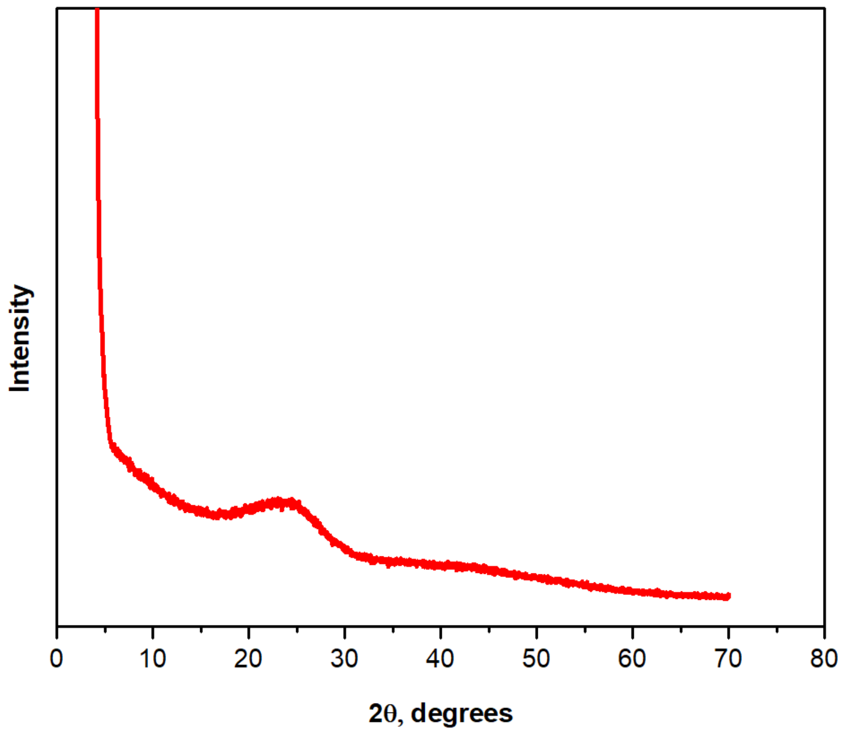

Figure S2. PXRD pattern profile of TATHCCP**.**

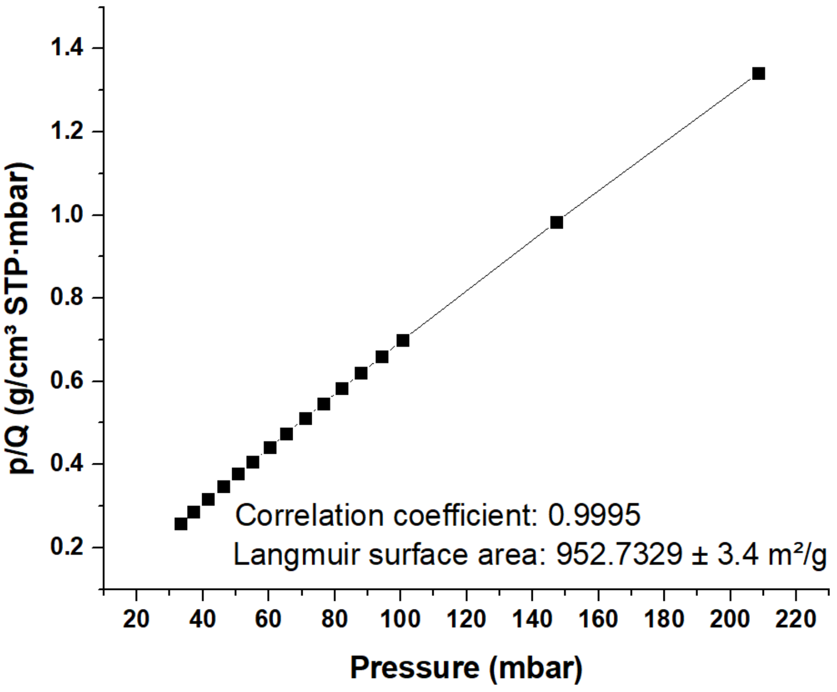

Figure S3. Langmuir surface area plot for TATHCCP calculated from the isotherm.

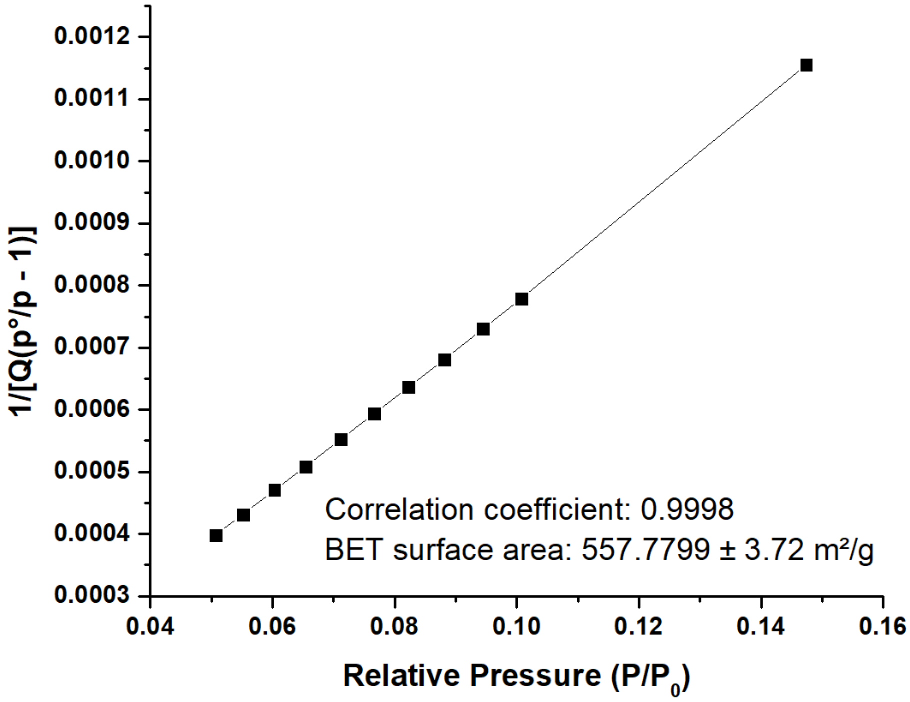

Figure S4. BET surface area plot for TATHCCP calculated from the isotherm.

Table S1. Comparable gas adsorption capacity of the TATHCCP with TATHCP.

Gas/Temperature	273 (K)	298(K)	323(K)
CO_2 wt %	9 / 12.55	5.5 / 7.68	3.2 / 4.75
CH_4 wt %	1.06 / 1.56	0.62 / 0.90	0.37 / 0.60
O_2 wt %	0.61 / 0.88	0.36 / 0.48	0.22 / 0.31
CO wt %	0.75 / 1.04	0.43 / 0.58	0.26 / 0.38
N_2 wt %	0.52 / 0.92	0.30 / 0.03	0.18 / 0.02
H_2 wt % (77K)	0.84 / 1.30	-	-

Table S2. Comparable results of gas selectivity for TATHCCP with TATHCP.

Gases/ Temperature	273K	298K	323K
CO_2/N_2 (05/95)	49.2	25.9	18.2
CO_2/N_2 (15/85)	50.0 / 38.4	25.9 / 22.3	18.3 / 15.3
CO_2/N_2 (50/50)	59.1 / 37.5	28.5 / 24.1	18.3 / 15.6
CO_2/CH_4 (50/50)	12.3 / 7.8	5.4 / 5.2	4.4 / 4.2
CO_2/CH_4 (5/95)	9.7 / 7.9	5.9 / 4.8	4.5 / 4.1
CO_2/O_2 (50/50)	45.9 / 40.6	23.5 / 25.5	16.4 / 18.0
CO_2/CO (50/50)	35.0 / 32.1	17.6 / 18.6	12.3 / 13.2

Table S3. Comparison of different microporous materials with respect to their textural and CO_2 uptake (wt % - mmol) values.

Material	BET (m^2/g^{-1})	CO_2 uptake (wt %- mmol) at 273K	CO_2 uptake (wt %/ mmol) at 298K
TATHCCP	557	9 / 2.1	5.5 / 1.27
TATHCP[1]	957	12.6	7.7
YBN-CC[2]	579	8.78 / 2.0	5.61 / 1.27
YBN-DMM[2]	784	12.70 / 2.87	7.70 / 1.75
YBN-DMB[2]	968	12.74 / 2.87	7.41 / 1.68
PAF-1[3]	5600	9.1	-
PON-1[4]	1400	10.8	-
TPI-1[5]	809	10.7	-
BPL carbon	-	9.15	-
IN[4]	243	7.5	4.85
BT[4]	571	10.6	6.4
BF[4]	1022	10.6	6.0
PECONF-2[6]	637	12.5	8.7
PECONF-4[6]	-	0.6	7.9
BLP-1H at [7]	-	7.4	-
COF-103[8]	-	7.6	-
CMP-1[8]	-	9.02	-
CBZ[4]	391	9.2	6.02
DBT[4]	493	9.7	6.06
BILP-1[13]	1172	4.27mmol	2.97mmol
BILP-2[13]	708	3.39mmol	2,36mmol
BILP-3[13]	1306	5.11mmol	3.29mmol
BILP-5[13]	599	2.9mmol	1.97mmol
ALP-1[25]	1235	5,36mmol	3.24mmol
ALP-4[25]	862	3.5mmol	1.84mmol

Table S4. Comparison of different microporous materials with respect to their H_2 uptake. (wt %) values.

Material	H_2 uptake (wt%) at 77K
TATHCCP	0.84
TATHCP[1]	1.30
YBN-CC[2]	1.59
YBN-DMM[2]	1.23
YBN-DMB[2]	1.18
Polyaniline[16]	0.85
CMP-2[17]	0.91
COF-103[8]	1.25

Table S5. Comparison of different microporous materials with respect to their CH_4 uptake (wt %) values.

Material	CH_4 uptake (wt%) at 273K
TATHCCP	1.06
TATHCP[1]	1.56
YBN-CC[2]	1.08
YBN-DMM[2]	1.61
YBN-DMB[2]	1.47
DBF[4]	1.47
BT[4]	1.34
BF[4]	1.22
CBZ[4]	1.16
IN[4]	0.73
MaSOF-1[10]	0.98
SBICC[11]	0.98
mesoPOF-1[12]	1.49
BILP-5[13]	1.77
BILP-10[14]	1.61
CC2[15]	1.82

Table S6. Comparison of different microporous materials with respect to their CO_2/N_2 (15/85) selectivity values at 273 K.

Material	Selectivity
TATHCCP	50
TATHCP[1]	38.4
YBN-CC[2]	60.0
YBN-DMM[2]	44.6
YBN-DMB[2]	159.1
FCBZz[18]	28.9
FCTCz[18]	26.1
TATCOF2[19]	5.9
YPTPA[20]	17.3
SPTPA[20]	30.6
TBPIM33[21]	18.1
TBPIM25[21]	17.0
PPF-1[22]	14.5
PPF-2[22]	15.4
PPF-3[22]	20.4
PPF-4[22]	15.0

Table S7. Comparison of different microporous materials with respect to their CO_2/CH_4 (50/50) selectivity values at 273 K.

Material	Selectivity
TATHCCP	9.7
TATHCP[1]	7.8
YBN-CC[2]	7.9
YBN-DMM[2]	6.9
YBN-DMB[2]	7.3
FCBZ[18]	5.8
FCTCz[18]	5.2
CMPs[23]	4
Cz-POF1[24]	4.4
Cz-POF3[24]	4.7

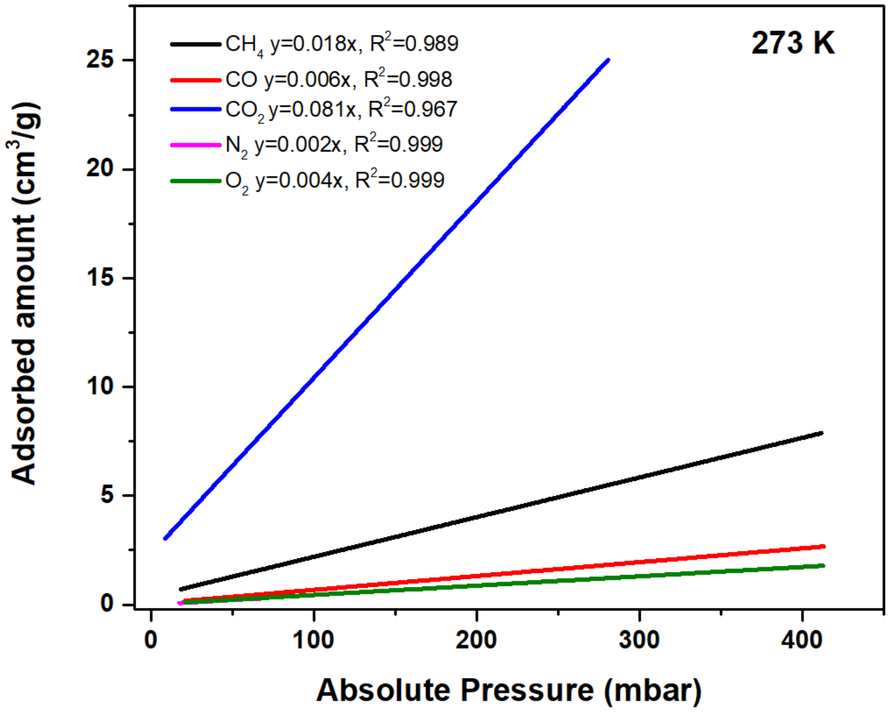

Figure S5. Initial slope fitting for gases of TATHCCP at 273K

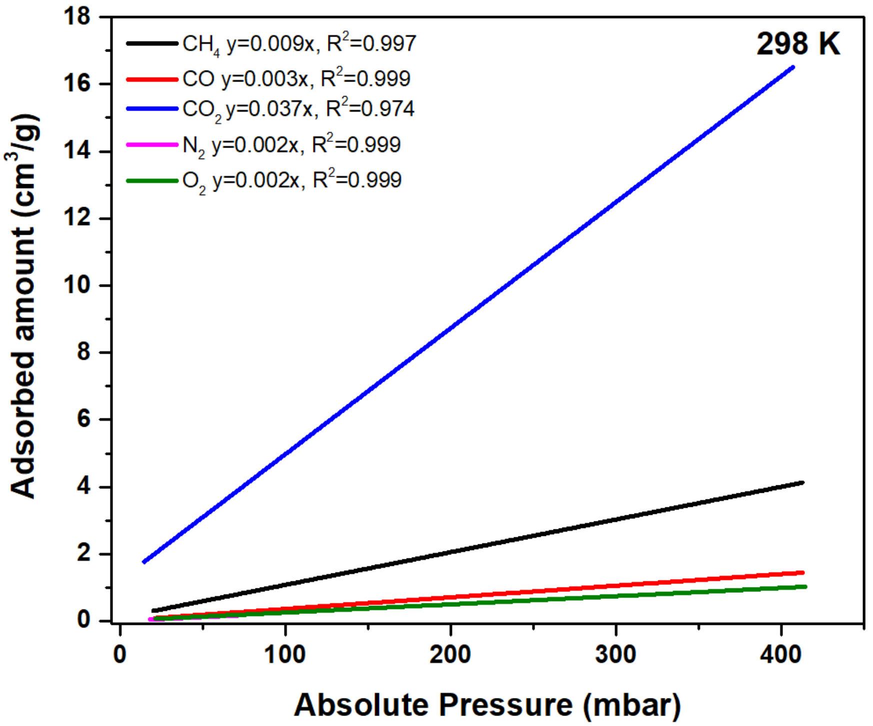

Figure S6. Initial slope fitting for gases of TATHCCP at 298K.

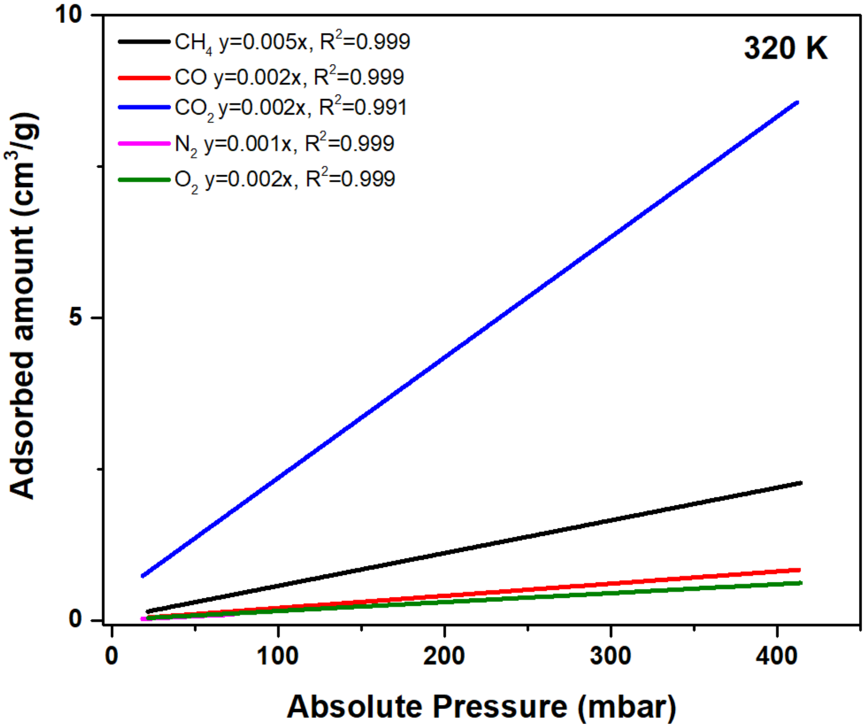

Figure S7. Initial slope fitting for gases of TATHCCP at 320 K.

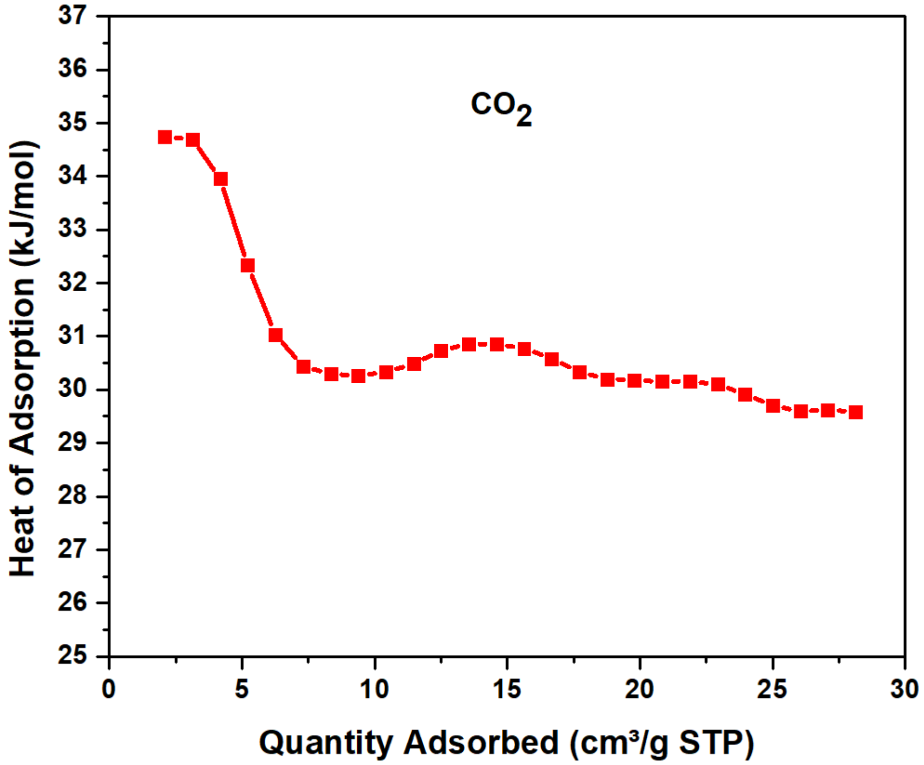

Figure S8. The isosteric heat of adsorption (Q_{st}) of TATHCCP for CO_2.

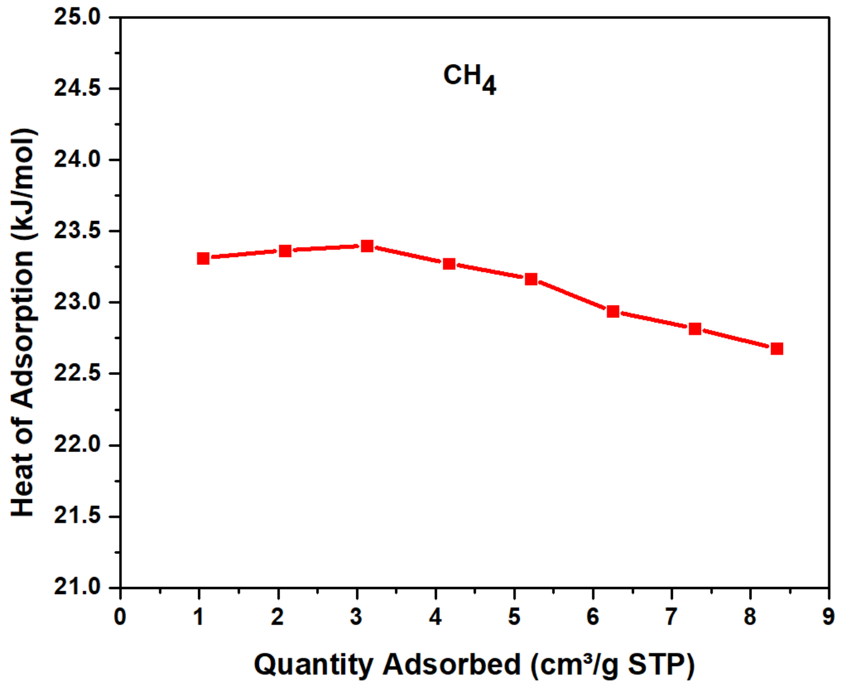

Figure S9. The isosteric heat of adsorption (Q_{st}) of TATHCCP for CH_4.

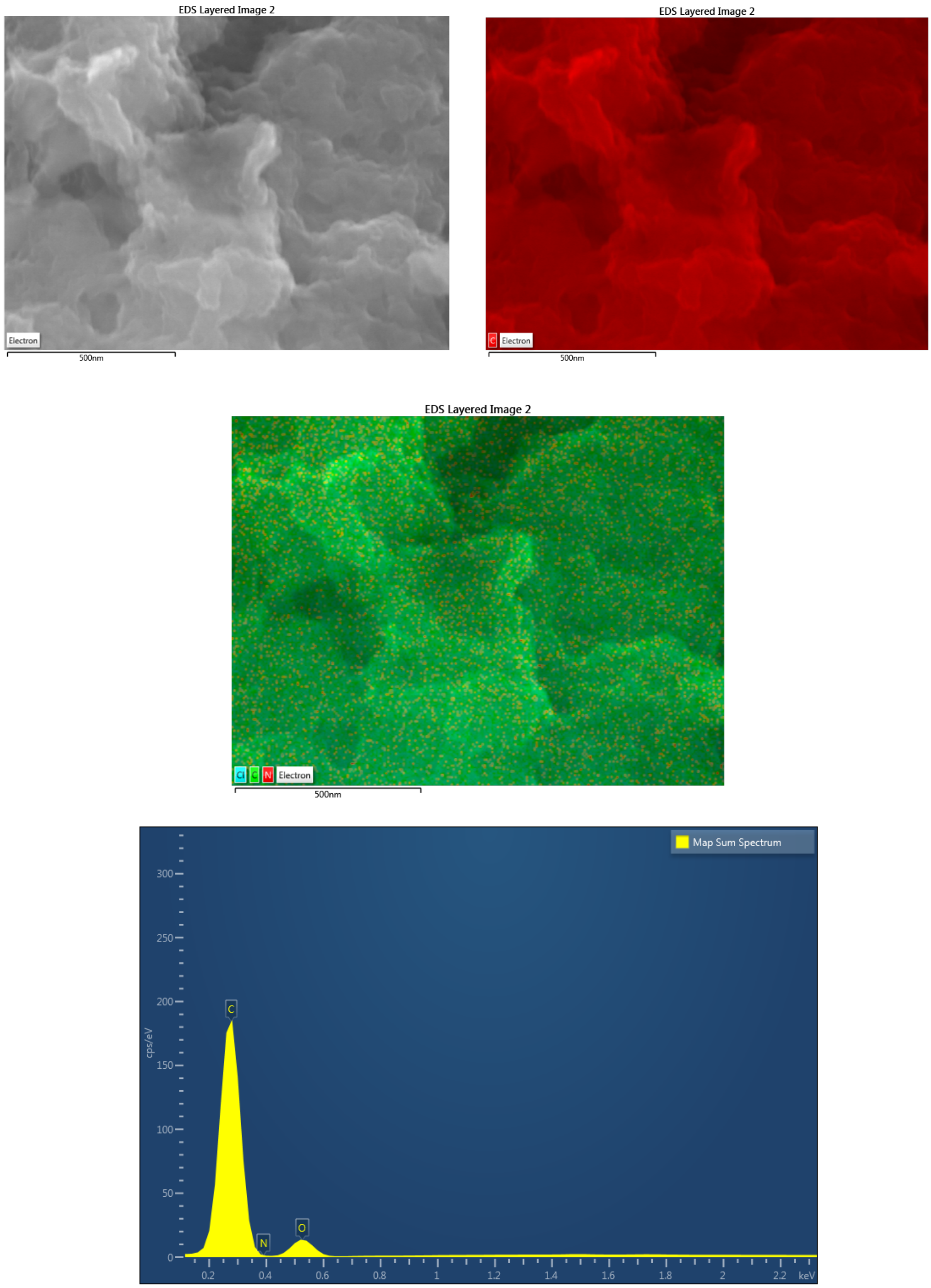

Figure S10. EDS spectra of TATHCCP.

4. Scanning electron microscopy (SEM) images of TATHCCP

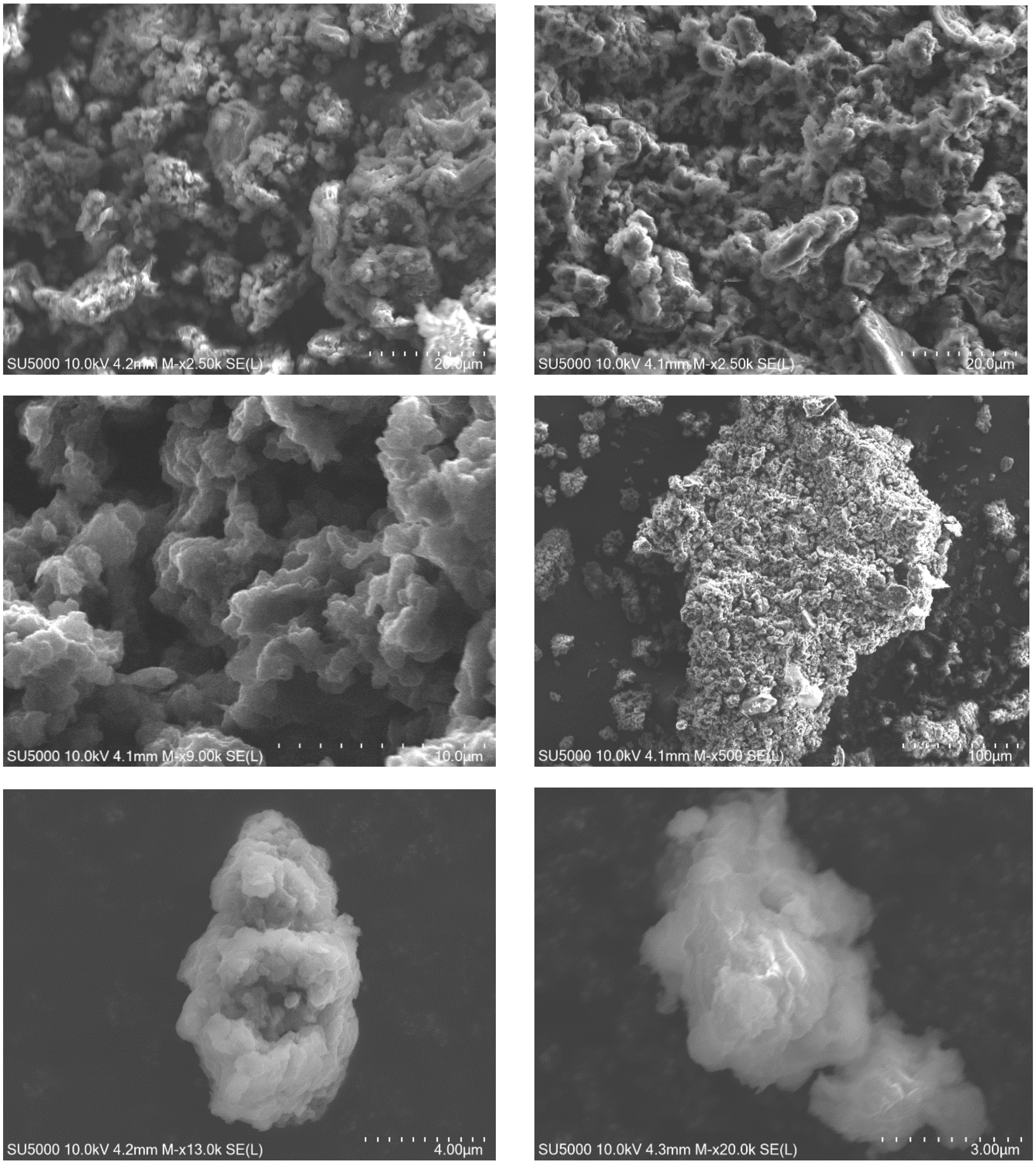

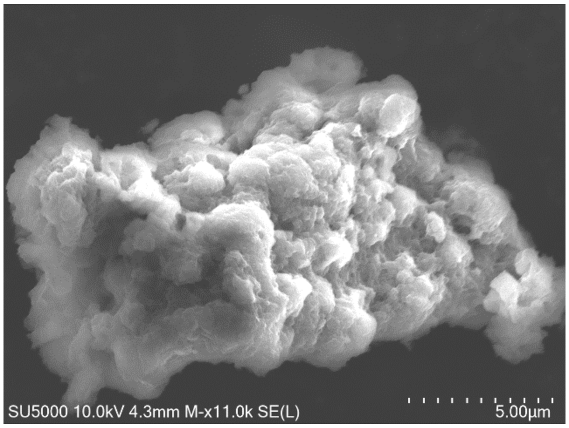
SU5000 10.0kV 4.3mm M-x11.0k SE(L)
5.00µm

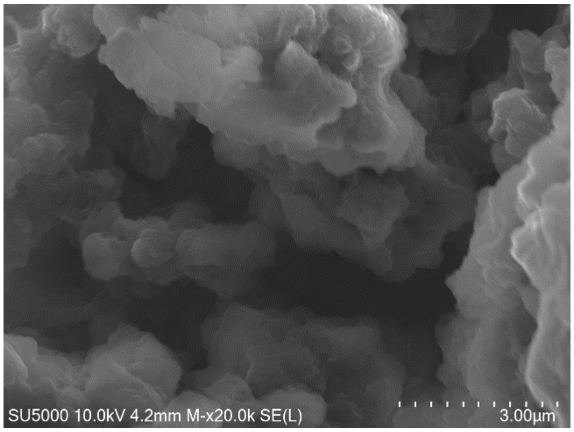
SU5000 10.0kV 4.2mm M-x20.0k SE(L)
3.00µm

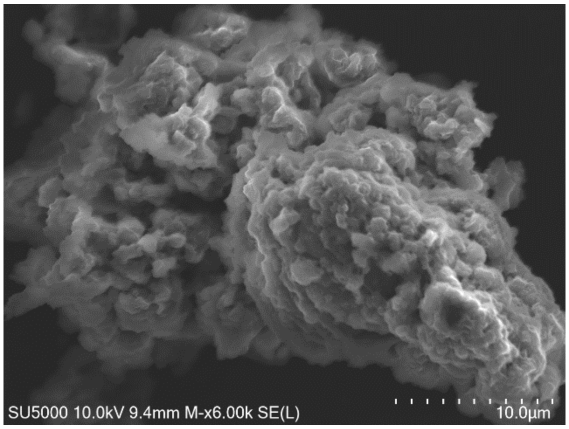
SU5000 10.0kV 9.4mm M-x6.00k SE(L)
10.0µm

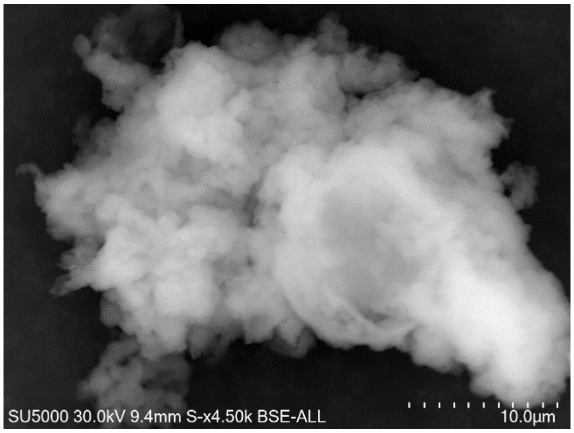
SU5000 30.0kV 9.4mm S-x4.50k BSE-ALL
10.0µm

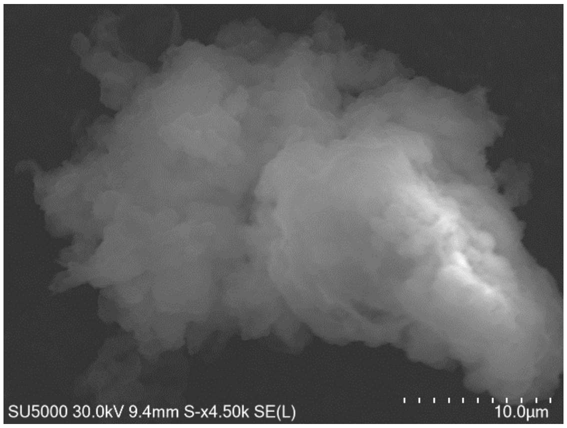
SU5000 30.0kV 9.4mm S-x4.50k SE(L)
10.0µm

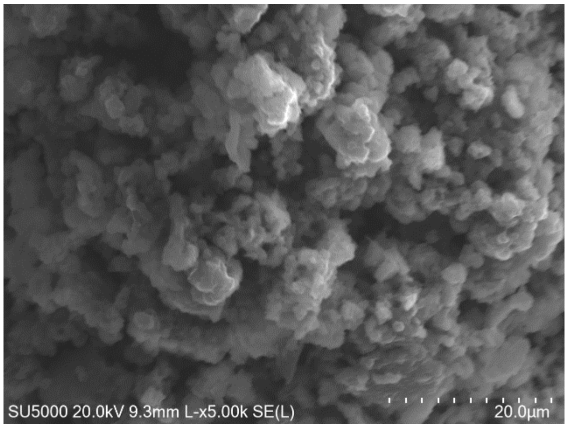
SU5000 20.0kV 9.3mm L-x5.00k SE(L)
20.0µm

5. References

1. Sadak AE, Karakuş E, Chumakov YM, Dogan NA, Yavuz CT. Triazatruxene-based ordered porous polymer: high capacity CO2, CH4, and H2 capture, heterogeneous Suzuki–Miyaura catalytic coupling, and thermoelectric properties. ACS Applied Energy Materials 2020; 3: 4983-4994.
2. Sadak AE. A comparative gas sorption study of dicarbazole-derived microporous hyper-crosslinked polymers. Microporous and Mesoporous Materials 2021; 311: 110727.
3. T. Ben, C. Pei, D. Zhang, J. Xu, F. Deng X et al. Gas storage in porous aromatic frameworks (PAFs). Energy Environmental Science 2011, *4*, 3991-3999.
4. Jeon HJ, Choi JH, Lee Y, Choi KM. Park JH, Kang JK. Highly selective CO2-capturing polymeric organic network structures. Advanced Energy Materials 2012, *2*, 225-228.
5. Liebl M. R., Senker J. Microporous Functionalized Triazine-Based Polyimides with High CO2 Capture Capacity. Chemistry of Materials 2013, *25*, 970-980.
6. Mohanty P, Kull LD, Landskron K. Porous covalent electron-rich organonitridic frameworks as highly selective sorbents for methane and carbon dioxide. Nature Communications 2011,*2*, 401-406.
7. Jackson KT, Rabbani MG, Reich TE, El-Kaderi HM; Synthesis of highly porous borazine-linked polymers and their application to H2, CO2, and CH4 storage. Polymer Chemistry 2011, *2*, 2775-2777.
8. Furukawa H, and Yaghi OM. Storage of hydrogen, methane, and carbon dioxide in highly porous covalent organic frameworks for clean energy applications. Journal of the American Chemical Society 2009, 131, 8875–8883.
9. Dawson R, Adams DJ, Cooper AI. Chemical tuning of CO2sorption in robust nanoporous organic polymers. Chemical Science 2011, 2, 1173–1177.
10. Mastalerz M, Hauswald HJS, Stoll R. A shape-persistent exo-functionalized [4 + 6] iminecage compound with a very high specific surface area. Chemical Communications 2012, 48, 130-132.
11. Germain J, Svec F, Fréchet JMJ. Preparation of Size-Selective Nanoporous Polymer Networks of Aromatic Rings: Potential Adsorbents for Hydrogen Storage. Chemistry of Materials 2008, *20*, 7069-7076.
12. Katsoulidis AP, Kanatzidis MG. Mesoporous hydrophobic polymeric organic frameworks with bound surfactants. selective adsorption of C2H6 versus CH4. Chemistry of Materials 2012, 24, 471-479.
13. Rabbani MG, El-Kaderi HM. Synthesis and characterization of porous benzimidazole-linked polymers and their performance in small gas storage and selective uptake. Chemistry of Materials 2012; 24: 1511-1517.
14. Rabbani MG, Sekizkardes AK, El-Kadri OM, Kaafarani BR, El-Kaderi HM. Pyrene-directed growth of nanoporous benzimidazole-linked nanofibers and their application to selective CO2 capture and separation. Journal of Materials Chemistry 2012, 22: 25409-25417.
15. Tozawa T, Jones JT, Swamy SI, Jiang S, Adams DJ et al. Porous organic cages. Nature materials 2009; 8(12), 973-978.
16. Germain J, Svec F, Fréchet JM. Preparation of size-selective nanoporous polymer networks of aromatic rings: potential adsorbents for hydrogen storage. Chemistry of Materials 2008, 20(22), 7069-7076.
17. Jiang JX, Su F, Trewin A, Wood CD, Niu H et al. Synthetic control of the pore dimension and surface area in conjugated microporous polymer and copolymer networks. Journal of the American Chemical Society 2008, 130(24), 7710-7720.
18. Yang X, Yu M, Zhao Y, Zhang C, Wang X et al. Hypercrosslinked microporous polymers based on carbazole for gas storage and separation. RSC Advances 2014, 4(105), 61051-61055.
19. Xie YF, Ding SY, Liu JM, Wang W, Zheng QY. Triazatruxene based covalent organic framework and its quick-response fluorescence-on nature towards electron rich arenes. Journal of Materials Chemistry C 2015, 3(39), 10066-10069
20. Yang X, Yao S, Yu M, Jiang JX. Synthesis and gas adsorption properties of tetra-armed microporous organic polymer networks based on triphenylamine. Macromolecular Rapid Communications 2014, 35(8), 834-839.
21. Wang ZG, Liu X, Wang D, Jin J. Tröger's base-based copolymers with intrinsic microporosity for CO 2 separation and effect of Tröger's base on separation performance. Polymer Chemistry 2014, 5(8), 2793-2800.
22. Zhu Y, Long H, Zhang W. Imine-linked porous polymer frameworks with high small gas (H2, CO2, CH4, C2H2) uptake and CO2/N2 selectivity. Chemistry of Materials 2013, 25(9), 1630-1635.
23. Qiao S, Du Z, Yang R. Design and synthesis of novel carbazole–spacer–carbazole type conjugated microporous networks for gas storage and separation. Journal of Materials Chemistry A 2014, 2(6), 1877-1885.

24. Chen Q, Luo M, Hammershøj P, Zhou D, Han Y et al. Microporous polycarbazole with high specific surface area for gas storage and separation. Journal of the American Chemical Society 2012, 134(14), 6084-6087.

25. Arab P, Rabbani MG, Sekizkardes AK, İslamoğlu T, El-Kaderi HM. Copper (I)-catalyzed synthesis of nanoporous azo-linked polymers: impact of textural properties on gas storage and selective carbon dioxide capture. Chemistry of Materials 2014, 26(3), 1385-1392.
